# An *In Silico* Approach for Modelling T-Helper Polarizing iNKT Cell Agonists

**DOI:** 10.1371/journal.pone.0087000

**Published:** 2014-01-31

**Authors:** Anton De Spiegeleer, Evelien Wynendaele, Matthias Vandekerckhove, Sofie Stalmans, Maxime Boucart, Nele Van Den Noortgate, Koen Venken, Serge Van Calenbergh, Sandrine Aspeslagh, Dirk Elewaut

**Affiliations:** 1 Laboratory for Molecular Immunology and Inflammation, Department of Rheumatology, Faculty of Medicine and Health Sciences, Ghent University, Ghent, Belgium; 2 Drug Quality and Registration (DruQuaR) Group, Department of Pharmaceutical Analysis, Faculty of Pharmaceutical Sciences, Ghent University, Ghent, Belgium; 3 Geriatrics Unit, Department of Internal Medicine, Faculty of Medicine and Health Sciences, Ghent University, Ghent, Belgium; 4 Laboratory of Medicinal Chemistry, Department of Pharmaceutics, Faculty of Pharmaceutical Sciences, Ghent University, Ghent, Belgium; Institut Pasteur, France

## Abstract

Many analogues of the glycolipid alpha-galactosylceramide (α-GalCer) are known to activate iNKT cells through their interaction with CD1d-expressing antigen-presenting cells, inducing the release of Th1 and Th2 cytokines. Because of iNKT cell involvement and associated Th1/Th2 cytokine changes in a broad spectrum of human diseases, the design of iNKT cell ligands with selective Th1 and Th2 properties has been the subject of extensive research. This search for novel iNKT cell ligands requires refined structural insights. Here we will visualize the chemical space of 333 currently known iNKT cell activators, including several newly tested analogues, by more than 3000 chemical descriptors which were calculated for each individual analogue. To evaluate the immunological responses we analyzed five different cytokines in five different test-systems. We linked the chemical space to the immunological space using a system biology computational approach resulting in highly sensitive and specific predictive models. Moreover, these models correspond with the current insights of iNKT cell activation by α-GalCer analogues, explaining the Th1 and Th2 biased responses, downstream of iNKT cell activation. We anticipate that such models will be of great value for the future design of iNKT cell agonists.

## Introduction

iNKT cells are a regulatory type of T cells that have been involved in many different disease settings. They express a T cell receptor that is composed of an invariant alpha-chain (Vα14-Jα18 in mice, Vα24-Jα18 in humans) and a restricted set of beta-chains (Vβ7, Vβ8.1, Vβ8.2 and Vβ2 in mice, Vβ11 in humans). These semi-invariant T cell receptors (TCR) recognize antigens in the context of CD1d, which is a non-classical MHC molecule expressed by antigen presenting cells (APCs). In contrast to classical MHC molecules, CD1d presents glycolipids instead of peptides. Upon TCR recognition, iNKT cells are activated which results in the production of large amounts of pro-inflammatory Th1- (IFN-γ, TNF-α) and anti-inflammatory Th2-cytokines (IL-4, IL-13) [1] both by iNKT cells themselves and activated bystander cells. Depending on the elicited cytokine response, glycolipid induced iNKT cell activation is able to alter the outcome of several pathologies, as has been observed in an experimental model of rheumatoid arthritis (CIA) [2] and different cancer models [3], making glycolipids promising therapeutics for immunomodulation [4]–[6]. Poor efficacy in some clinical trials is attributed to the opposing activities of simultaneous secreted Th1 and Th2 cytokines. Hence, glycolipids capable of inducing a biased Th1 or Th2 response are believed to afford superior clinical effectiveness.

In the physiological process of ageing, iNKT cell changes are observed [7] and a Th1/Th2 imbalance in favour of Th2 dominance is known as one of the most important detrimental shifts of immune senescence [8]–[11].

Alpha-galactosylceramide (α-GalCer), the synthetic prototype of an iNKT cell activating glycolipid, consists of a galactose connected to a lipid backbone by an alpha-glycosidic binding. The lipid backbone consists of a ceramide: an N-acyl chain coupled to a phytosphingosine-chain. Alpha-galactosylceramide evokes the production of a combined Th1/Th2-cytokine response [1].

Currently, different hypotheses about how a specific iNKT cell agonist can induce a polarized Th1 or Th2 response are being intensively examined but it has been shown already that a reasonable strategy is to alter the structure of the glycolipids. This bias or selectivity should lead to more disease-specific therapies. Nowadays, hundreds of these altered glycolipids have been synthesized and tested on their ability to provoke different cytokine-responses, in mice as well as in humans, *in vitro* and *in vivo*. Despite the numerous chemical and immunological data reported, no overall combined *in silico* analysis is up till now available. In this study, we used a system biology computational approach exploring the chemical and immunological spaces wherein the currently known iNKT cell activators are situated. All available existing data (up to December 2012), as well as new results from novel analogues (from our research group), were included. The chemical space of the iNKT cell activating glycolipids is defined by their chemical properties, quantitatively expressed by descriptors, while the immunological space is defined by different cytokine-responses. This novel approach resulted in an *in silico* structure-immune model of iNKT-activators. We believe such a model could be valuable for future design of new compounds, leading to optimized iNKT cell based therapies.

## Materials and Methods

### Dataset

We searched Web of Science, using the keywords ‘α-GalCer-analogues’ and ‘iNKT cell activators’ till the end of 2012. We only included articles containing defined chemical structures accompanied with quantitative immunological results. After a preliminary search, we found that the most frequently used immunological markers are the cytokines IL-2, IFN-γ, IL-4 and IL-13. Moreover, we could define five test-models: *mice/in vivo, mice/in vitro/cell-cell, mice/in vitro/cell-plate, human/in vitro/cell-cell and human/in vitro/cell-plate*, where the cell-cell system refers to CD1d and iTCR both expressed on cells, while for the cell-plate system, CD1d is plate-bound. As such, a maximum of 20 immunological responses for every analogue is thus possible. We used the cytokine-values reported in the articles. We tested some new analogues with slightly different composition as well as published analogues, to expand the chemical and immunological space of the dataset and improve our models. These new analogues can grossly be divided in three groups: substitutions on the 6-OH of a galacturonic acid sugar (**138**, **139**, **140**, **142**, **143** and **145**), amine substitutions of the OH-backbone of the sphingosine chain (**154**, **155**, **156**, **157** and **159**) and variations on the length and phenylation of the lipid chains (**160**, **161**, **163**, **164** and **165**). To account for the variability in the immunological responses from different articles and experiments, the immunological response of the analogue is normalized to the response of α-GalCer, which is numbered as compound **1**. This relative immunological response, *i.e.* the ratio relative to the reference compound **1,** is the immunological result used in the computations. If different doses or incubation times for one analogue were used in the same experiment, only those results are withheld corresponding to the dose and incubation time for which α-GalCer gave maximal cytokine-response.

In total, we analyzed 333 analogues (*Supporting information S1*) from 57 articles (*Supporting information S2*) and from unpublished, new data of our lab.

### Chemical space

With the use of the chemical software programs Hyperchem 8.0.8 (Hypercube, Gainesville, USA) and Dragon 5.5 (Talete, Milan, Italy), the three-dimensional structure of the 333 glycolipids was optimized, followed by the calculation of 3228 chemical descriptors. After removal of the constant descriptors, a final dataset of 1656 descriptors was retained. Multivariate data-analysis on this resulting 333×1656 data-matrix was performed using Principal Component Analysis (PCA) and Hierarchical Cluster Analysis (HCA) with SIMCA-P+ 12.0 (Umetrics, Sweden) and SPSS Statistics 20.0 (Illinois, USA) software programs, respectively. PCA and HCA are two different ways to cluster molecules based on their chemical properties as quantified by their descriptors. See *Supporting information S3* for a more in-depth description [12], [13].

### Immunological space

For the analysis of frequencies, variability and correlations, SPSS Statistics 20.0 was used. In order to integrate the different cytokine responses into a limited set of physiological interesting responses, a multi-criteria decision technique was employed, using Derringer’s concept of desirability [14], [15], [16]. Every immunological response was first linearly transformed into a dimensionless desirability (d) value, ranging from 0.1 to 0.9, where 0.1 is the least and 0.9 the most desired cytokine response (*Supporting information S4 and S5*). As such, analogues with a high Th1 desirability should induce high IFN-γ responses – high IFN-γ values get d-values close to 0.9 – while they induce low IL-4 responses – low IL-4 values get d-values close to 0.9. The opposite is true for analogues with a high Th2 desirability. These standardized d-values were combined into a global D-value for each analogue, which is the geometric mean. The analogue with the highest D-value expresses the best overall combination of the different desired responses. Inherent to this desirability concept, all D-values lie between 0.1 and 0.9. Analogues where both the IFN-γ and IL-4 responses are very high or very low are not of interest for fine-tuning the immune system in a clinical setting, and this is reflected in a low D-value of 0.3 (geometric mean of 0.1 and 0.9). We calculated four different D-values for each analogue where sufficient information was available: Th1 *in vivo*, Th2 *in vivo*, Th1 *in vitro* and Th2 *in vitro*.

### Structure-immune modelling

Stepwise linear regression (Multiple Linear Regression, MLR) and Partial Least Squares (PLS) regression were performed for the modelling using SPSS Statistics 20.0 and SIMCA-P+ 12.0 software programs [17]. To obtain the MLR models, F values to enter/remove were set at 0.05 and 0.10, respectively. The models were also validated for their ability to predict the quantitative desirability value of newly synthesized analogues. Therefore, a PLS seven-fold internal cross-validation was performed, resulting in a cross-validated R^2^ and Q^2^ value, which is an estimate of the predictive ability of the model. To visualize the goodness-of-fit of the models to categorize the analogues as strong or weak Th1 or Th2 molecules, an ROC curve was built. Strong analogues have a D-value higher than α-GalCer. The ‘goodness’ of the ROC curve was expressed as the area under the curve.

## Results and Discussion

### Dataset

*Table 1* gives the distribution of our dataset, ordered by the different test-systems and markers used. In total, 851 results were available, covering 333 different α-GalCer analogues. Some α-GalCer analogues were evaluated by different research groups using multiple methods; for these analogues, the normalized data obtained with a specific test-system and marker were averaged to obtain one result as represented in the total set of 851 results.

**Table 1:** Distribution of methodologies used in α-GalCer immunological studies.

Test-model	Marker	Number of results	%
*Mice/in vivo*	IL-2	3	0.35
	IFN-γ	120	14.10
	IL-4	113	13.28
	IL-13	1	0.12
*Mice/in vitro/cell-cell*	IL-2	87	10.22
	IFN-γ	77	9.05
	IL-4	67	7.87
	IL-13	13	1.53
*Mice/in vitro/cell-plate*	IL-2	66	7.76
	IFN-γ	1	0.12
	IL-4	1	0.12
	IL-13	1	0.12
*Human/in vitro/cell-cell*	IL-2	19	2.23
	IFN-γ	121	14.22
	IL-4	96	11.28
	IL-13	56	6.58
*Human/in vitro/cell-plate*	IL-2	5	0.59
	IFN-γ	2	0.24
	IL-4	1	0.12
	IL-13	1	0.12
Total		851	100

The test-system most frequently applied to obtain our dataset is the *human/in vitro/cell-cell* system (34.31%). This is followed by two test-systems almost equally applied: the *mice/in vitro/cell-cell* (28.67%) and *mice/in vivo* method (27.85%). The two *in vitro/cell-plate* methods are only marginally used, with the *human/in vitro/cell-plate* method only reported in two studies (*S2: references 4 and 57*). Therefore, unless otherwise mentioned, with the *in vitro* test-models is meant the in *vitro/cell-cell* test-models in this article. IFN-γ and IL-4 are the markers most frequently measured in the three most important test-systems, *i.e.* their frequencies ranging from 7.87% to 14.22%. The IL-2 data were mostly obtained from the *mice/in vitro/cell-cell* method (10.22%), while IL-13 was only used to a limited extent in the *human/in vitro/cell-cell* test-system (6.58%).

### Chemical space

**Figure 1:**
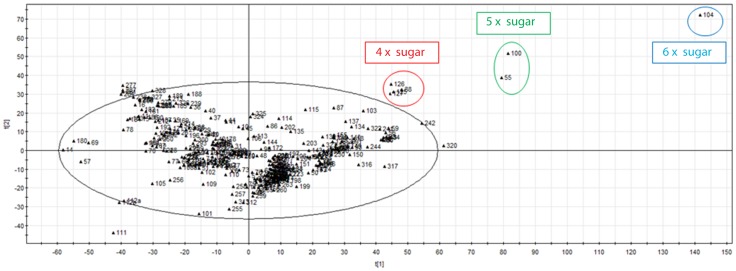
PCA score plot with the two first principal component vectors t(1) and t(2). Each PCA vector represents a specific combination of the 1656 chemical descriptors. The three most outlying groups can be observed. An in depth study was performed with HCA analysis.

The PCA description is given in *Supporting information S6*. Looking at the score plot (*Figure 1*), three outlying groups can be observed, mainly based on the sugar composition: the molecules containing four (**125**–**127** and **88**), five (**100** and **55**) and six (**104**) sugar-derived moieties are classified in different groups. An in-depth study of the different clusters was performed using the dendrogram of the HCA analysis (*Supporting information S7*). This visual representation categorizes the α-GalCer analogues containing two or three sugar molecules (**56**, **58**, **59**, **79**–**84**, **86**, **87**, **89**–**93**, **103**, and **230**) in one cluster. This chemical segregation can be linked to functional segregation with previous studies showing that the extra sugar groups have to be accommodated by the iNKT TCR, resulting in loss of energy and thus weaker antigens [18]. The further hierarchical clustering results (dendrogram) are described in *Supporting information S8*.

### Immunological space

Before transforming the immunological responses in dimensionless desirability (d) values for the structure-immune modelling, the biological data were investigated with the objective of (1) evaluating the value of each test-model, and (2) examining the currently assumed relations between IFN-γ, IL-4 and IL-13.

**Figure 2:**
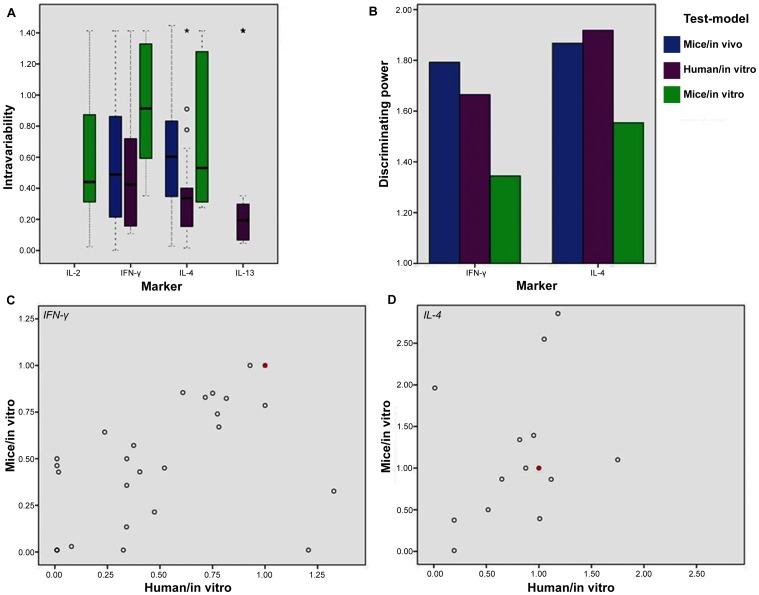
Comparison of the different test-models. (A) Intra-variability. Box-plots of relative standard deviations are shown. Each value represents a compound with its relative standard deviation. The human test-model shows a lower median than the mice test-models, which means that in the human test-model more compounds have uniform cytokine-responses between different research groups (IL-4 p = 0.015, IFN-γ p = 0.13, Kruskal-Wallis test). (B) Inter-variability. The height of the bars represents the discriminating power of a specific test-model for a specific cytokine. This is calculated by a relative standard deviation of the biological responses in a specific test-model (corrected for the intrinsic intra-variability). (C + D) Relation between *mice/in vitro* and *human/in vitro* assay for (C) IFN-γ (ρ_s_ = 0.56, p = 0.002) and (D) IL-4 (ρ_s_ = 0.38, p = 0.18).

To gain more insight in the different test-models, we compared the intra-variability and the inter-variability of the analogues in the different test-models and looked at their relations. The intra-variability or intrinsic method variation was quantified by the relative standard deviation for every analogue that had identical biological responses from more than one study. In ideal conditions, the responses for the same analogue in different studies are equal, represented by a very low relative standard deviation. The available results are shown in *Figure 2A*, overall suggesting that IL-4 and IFN-γ variability are the lowest in the *human/in vitro* test-model. This means that *human/in vitro* test-models are more precise to uniformly compare IL-4 and IFN-γ biological activity of α-GalCer analogues than *mice/in vitro* or *mice/in vivo* test-models, where the variation intrinsic to the test-model is high. The inter-variability or variation between different analogues for a specific test-model is indicative for its discriminating power. In the worst-case scenario, the inter-variability for a given test-model is zero, which means that all the analogues give the same biological response and the test-model is non-discriminating. *Figure 2B* visualizes the inter-variability or discriminating power for IFN-γ and IL-4 in 3 different test-models. The IL-2 and IL-13 data were not available in sufficient amounts in different test-models for comparison and are therefore not included. From the relative standard deviation for IFN-γ and IL-4 (*Figure 2B*), it is observed that the *human/in vitro* test-model, similar to the *mice/in vivo* test-model, is more discriminating than the *mice/in vitro* test-model. This implies that, when similar analogues are to be fine-tuned in a differentiation study, preference should be given to the *human/in vitro* or *mice/in vivo* test-model when possible. Beside the superiority of the *human/in vitro* and *mice/in vivo* test-models in discriminating power, it is also observed that *mice* results are closely related to the *human* results for the *in vitro* test-systems (*Figure 2C-D*). The *mice/in vivo* test-model did not show any meaningful relation with the other systems (*Supporting information S9*): this test-model is thus delivering new information next to the *mice/in vitro* and *human/in vitro* test-models, which are giving rather similar information.

In conclusion, these three studied aspects of comparison imply that if one has to make test-model choices, the *mice/in vitro* test-model is becoming superfluous, while the *mice/in vivo* and the *human/in vitro* test-models are giving the most discriminative and orthogonal information.

**Figure 3:**
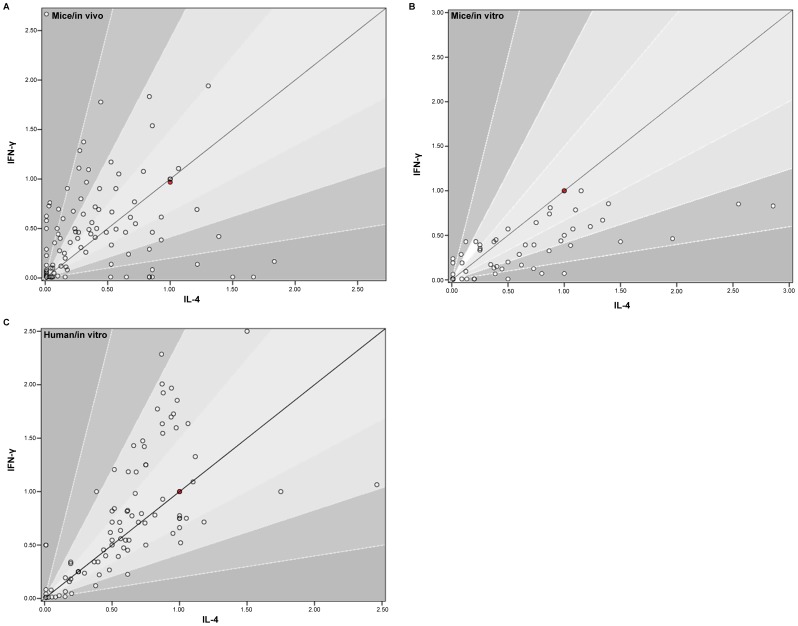
Th1/Th2 polarization in the different test-models. The graph has a color-gradient with the darker parts representing stronger polarizers. α-GalCer is shown in red on the y = x line.

When we plotted IFN-γ versus IL-4 in the three test-models under evaluation (*Figure 3*), it was interesting to see that stronger Th1 or Th2 polarized analogues existed in the *in vivo* test-model: quite some points are in the outer parts of the graph (*Figure 3A*). Much less outward points are observed with the *in vitro* test-models (*Figure 3B and 3C*). This can be explained by the relative lack of bystander cells in the *in vitro* test-models, such as NK-cells, which are supposed to play an important role in cytokine-polarization [19], [20]. Supporting this view is the fact that the cytokine-polarization is less with the *human/in vitro* model compared to the *mice/in vitro* model. We hypothesize that this is due to the frequent use of mice spleen-extracts in the *mice/in vitro* model, where other cells than iNKTs and APCs are also present. Beside the presence of bystander cells to explain the cytokine-polarization, a distinct APC profile *in vivo* versus *in vitro* could also play a role [21]. The position of the data-points in *Figure 3B* also implies that there is a Th2-overestimation with the *mice/in vitro* test-models compared to the other test-models.

The well-accepted association between the two Th2-cytokines IL-4 and IL-13 was also confirmed in the human and mice models by our data (*not shown*).

### Structure-immune modelling

**Figure 4:**
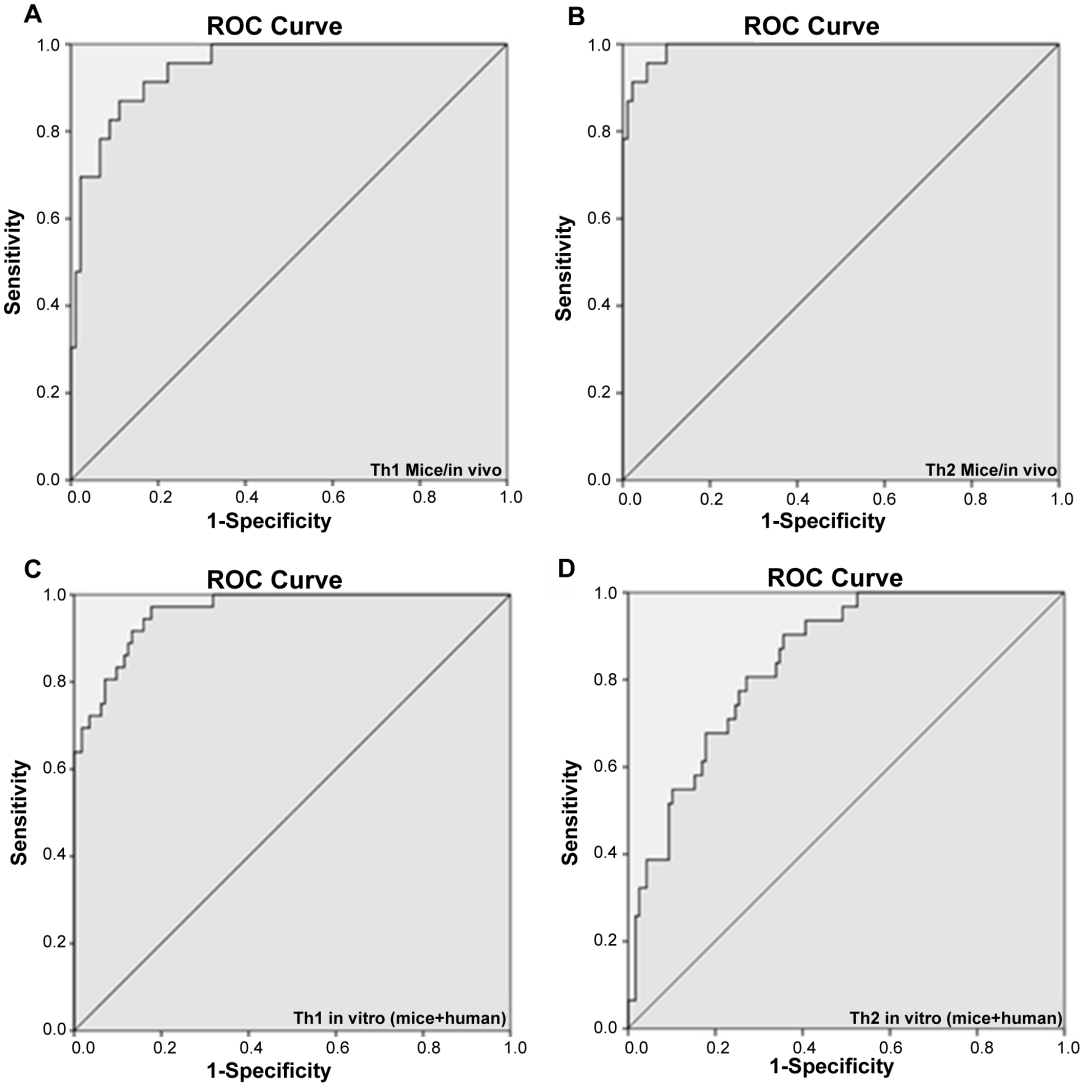
ROC curves. The AUC is an estimate of the goodness of fit. (A) Th1 *mice/in vivo* (AUC =  0.948). (B) Th2 *mice/in vivo* (AUC =  0.991). (C) Th1 *in vitro* (mice+human) (AUC = 0.961). (D) Th2 *in vitro* (mice+human) (AUC = 0.843). AUC: Area Under the Curve.

Based on both the chemical space and the immunological space, structure-immune models were computed. The immunological response variable of these models is represented by a D(esirability)-value, which is a combination of desired cytokine-values. For example, an analogue with high IFN-γ and low IL-4 values has a high Th1 D-value. The *human* and *mice in vitro* data were combined in one model because of a gain in sample size/power and the high correlation between these two test-systems (see *Immunological space*). *Supporting information S10* gives the chemical descriptors and their respective coefficients for both the *in vivo* and *in vitro* models. To validate our models, internal cross-validation was used, which involves repeatedly leaving a sample out of the model and test this sample in the model made by the remaining data. From the goodness-of-fit R^2^ values (0.64–0.96) as well as from the predictive Q^2^ values (0.51–0.78) (*S10*) can be derived that our structure-immune models well explain the variability observed. *Figure 4* gives a visual representation of the prediction-models in a ROC curve, where the dichotomic cut-off is set at the D-value of α-GalCer. This gives an idea about the power of our models to select analogues with stronger Th1 or Th2 response than α-GalCer. From these ROC curves, it can again be concluded that the Th1 *in vivo*, Th2 *in vivo* and Th1 *in vitro* models are excellent tests (AUC > 0.9) and the Th2 *in vitro* a good test (AUC > 0.8) to distinguish the strong α-GalCer analogues from the weak ones.

In our laboratory, we analysed 16 new analogues (**138**–**140**, **142**, **143**, **145, 154**–**157**, **159**–**161**, **163**–**165**) using *mice/in vivo* experiments and found only compounds **138** and **142**, with aromatic substitutions on the 6-OH of a galacturonic acid sugar, to be of further interest: these molecules had a higher Th1 *in vivo* desirability than α-GalCer **1**. Our computed model also indicated only these two analogues, *i.e.* **138** and **142,** as more desirable than α-GalCer **1**, which examplifies the good R^2^ and Q^2^ of our model.

Analyzing these regression models gives information about the chemical characteristics of the α-GalCer analogues which are important for their immunological behaviour. In literature, to explain the Th1/Th2 balance, it is demonstrated that a Th1 cytokine pattern requires longer TCR stimulation than a Th2 cytokine pattern [22]. Therefore the overall stability of the CD1d-glycolipid-TCR complex is often analyzed, including the stability of the glycolipid antigen *in vivo* [19], [22]–[25]. In the following section, this information is used to evaluate the calculated *in vivo* and *in vitro* models. The meaning of the chemical descriptors used in the text below is given in *Supporting Information S11*.

Our models (*S10)* suggest that strong Th2 analogues depend of many more chemical properties than Th1 analogues. For the *in vivo* models for example, a similar R^2^ is computed with more than double the amount of chemical descriptors needed to explain the Th2 versus Th1 model. With the *in vitro* data, we were not able to build an excellent Th2 model with a limited set of chemical descriptors, suggesting the dependence of many more chemical descriptors.

From the Th1 *in vivo* model (*e.g.* descriptors nROCON,, C-041 and Morxxe/m), it is clear that the presence of a carbamate or ureum linker is preferred for Th1 activity. This is in accordance with previous crystallographic data, demonstrating that the linker between the 6-OH galactose and the aromatic group is very important, affecting TCR affinity and thus antigenicity; the carbamate analogues analyzed in our lab (**129**–**135** and **195**) displayed a significantly stronger Th1 profile due to higher IFN-γ and IL-12 production compared to α-GalCer **1** [26], [27]. Next, the presence of a fluor atom is recommended for a high Th1 response as well: the PLS and MLR models both indicate many F-descriptive parameters, *e.g.* F-084 and Bx[C-F]. This is confirmed by molecules **134**, **137** and **325**, inducing comparable levels of IFN-γ as α-GalCer **1** with only marginal levels of IL-4; this effect is caused by a higher binding strength and stability with the TCR of iNKT cells [28].

The Th1 *in vitro* model is characterized by the presence of urea derived moieties (nCONN descriptor), similar to the Th1 *in vivo* model. This again complies with crystallographic results: the urea linker has an optimal length to induce the formation of a third small anchor (hydrophobic) pocket, as well as the correct spacing between the carbonyl oxygen and the galactose to form the conserved H-bond with Thr159, without significantly affecting the galactose orientation [26]. Next, modifications at the long N-acyl tail of the ceramide by (F-substituted) aromatic groups (nCb-, B09 [C-F] and autocorrelation descriptors evaluating the chain length) influence the Th1 activity as well: the stability of the GalCer-CD1d complex is increased due to additional hydrophobic forces between the aromatic goups and the A’-pocket, leading to higher Th1 activity levels. Phenyl modifications on the sphingosine moiety decrease the glycolipid flexibility, stabilizing the F’-pocket of the CD1d binding site and thereby increasing CD1d affinity for the iNKT TCR [28], [29].

With only these limited number of described structural characteristics, the high Th1 activity of already 10/22 (*in vivo*) and 27/35 (*in vitro*) α-GalCer analogues (activity > α-GalCer **1**) could be explained.

For high Th2 activity, *in vivo* or *in vitro*, aliphatic desaturations should be present (*e.g.* nR = Cs, Ui, nBM and nCconj descriptors, based on MLR and PLS models). This correlates with previous studies where increased hydrophilicity in the acyl or sphingosine chain, *e.g.* by the addition of desaturations (C20:2), was associated with a reduced stability of the GalCer-CD1d complex, leading to Th2 polarization and relatively weak iNKT cell activation [22]–[25]. The increase in Th2 activity by higher hydrophilicity is confirmed by *e.g.* the Mp, nHBonds and nOHp descriptors of the Th2 *in vitro* model. The high Th2 activity of molecule **99** (OCH), the prototypical Th2 biasing α-GalCer analogue with a truncated sphingosine chain and a reduced acyl chain [22], [23], can be explained by the negative coefficient values of the ECC (eccentricity) and W3D (shape) descriptors, favouring thus compact conformations. Next, 6”-triazole-substituted α-GalCer analogues were reported to exhibit a small Th2 cytokine-biasing response as well [30]. These findings also appear in both the Th2 *in vivo* and *in vitro* model, *e.g.* the Bx[N-N], Fx[N-N], nTriazoles, H-048, C-034 and N-073 descriptors. Interestingly, the C-041 descriptor (ureum/carbamate), which was found to be important for Th1 activity, shows a negative coefficient value in the Th2 *in vivo* model. This again confirms the Th1/Th2 biasing potential of urea and carbamate analogues.

About half of the molecules with high Th2 activity, *i.e.* Th2 activity higher than the activity of α-GalCer **1** (*in vivo*: 10/19; *in vitro*: 13/29) could be confirmed as potent Th2-biasing analogues, based on the limited characteristics described above.

We can thus conclude that our prediction models correspond well with the current binding hypotheses explaining the polarization of the cytokine profile after iNKT cell activation and that they have a good to excellent accuracy to identify α-GalCer analogues with strong polarizing properties. Therefore, we propose these novel models to be used in a high-throughput screening approach, decreasing analysis time and costs for functionality analysis.

## Supporting Information

File S1 **Structures with ID-number.**
(DOCX)

File S2 **References.**
(DOCX)

File S3 **Clustering methods.**
(DOCX)

File S4 **Derringer transformation.**
(DOCX)

File S5 **Maximally found response values Ymax.**
(DOCX)

File S6 **PCA model description.**
(DOCX)

File S7 **Dendrogram using Average Linkage.**
(TIF)

File S8 **Results of HCA-clustering.**
(DOCX)

File S9 **Mice/in vivo compared with other test-models.**
(TIF)

File S10 **MLR and PLS models.**
(DOCX)

File S11 **Meaning of described chemical descriptors.**
(DOCX)
