# Supplementary material for: An In Silico Approach for Modelling T-Helper Polarizing iNKT Cell Agonists
Source: PLoS One. 2014 Jan 31;9(1):e87000. doi: 10.1371/journal.pone.0087000 (PMC3909045; doi:10.1371/journal.pone.0087000)
Supplement: File S1 — Structures with ID-number. (DOCX) [file pone.0087000.s001.docx]

# Supporting information S1

**STRUCTURES WITH ID-NUMBER**.

| **ID** | **Chemical structure** | **ID** | **Chemical structure** |
| --- | --- | --- | --- |
| 1 |  | 16 |  |
| 2 |  | 17 |  |
| 3 |  | 18 |  |
| 4 |  | 19 |  |
| 5 |  | 20 |  |
| 6 |  | 21 |  |
| 7 |  | 22 |  |
| 8 |  | 23 |  |
| 9 |  | 24 |  |
| 10 |  | 25 |  |
| 11 |  | 26 |  |
| 12 |  | 27 |  |
| 13 |  | 28 |  |
| 14 |  | 29 |  |
| 15 |  | 30 |  |
| 31 |  | 45 |  |
| 32 |  | 46 |  |
| 33 |  | 47 |  |
| 34 |  | 48 |  |
| 35 |  | 49 |  |
| 36 |  | 50 |  |
| 37 |  | 51 |  |
| 38 |  | 52 |  |
| 39 |  | 53 |  |
| 40 |  | 54 |  |
| 41 |  | 55 |  |
| 42 |  | 56 |  |
| 43 |  | 57 |  |
| 44 |  | 58 |  |
| 59 |  | 73 |  |
| 60 |  | 74 |  |
| 61 |  | 75 |  |
| 62 |  | 76 |  |
| 63 |  | 77 |  |
| 64 |  | 78 |  |
| 65 |  | 79 |  |
| 66 |  | 80 |  |
| 67 |  | 81 |  |
| 68 |  | 82 |  |
| 69 |  | 83 |  |
| 70 |  | 84 |  |
| 71 |  | 85 |  |
| 72 |  | 86 |  |
| 87 |  | 100 |  |
| 88 |  | 101 |  |
| 89 |  | 102 |  |
| 90 |  | 103 |  |
| 91 |  | 104 |  |
| 92 |  | 105 |  |
| 93 |  | 106 |  |
| 94 |  | 107 |  |
| 95 |  | 108a |  |
| 96 |  | 108b |  |
| 97 |  | 109 |  |
| 98 |  | 110 |  |
| 99 |  | 111 |  |
| 112a |  | 124b |  |
| 112b |  | 124c |  |
| 113 |  | 124d |  |
| 114 |  | 125 |  |
| 115 |  | 126 |  |
| 116 |  | 127 |  |
| 117 |  | 128 |  |
| 118 |  | 129 |  |
| 119 |  | 130 |  |
| 120 |  | 131 |  |
| 121 |  | 132 |  |
| 122 |  | 133 |  |
| 123 |  | 134 |  |
| 124a |  | 135 |  |
| 136 |  | 149 |  |
| 137 |  | 150 |  |
| 138 |  | 151 |  |
| 139 |  | 152 |  |
| 140 |  | 153 |  |
| 141 |  | 154 |  |
| 142 |  | 155 |  |
| 143 |  | 156 |  |
| 144 |  | 157 |  |
| 145 |  | 158 |  |
| 146 |  | 159 |  |
| 147 |  | 160 |  |
| 148 |  | 161 |  |
| 162 |  | 175 |  |
| 163 |  | 176 |  |
| 164 |  | 177 |  |
| 165 |  | 178 |  |
| 166 |  | 179 |  |
| 167 |  | 180 |  |
| 168 |  | 181 |  |
| 169 |  | 182 |  |
| 170 |  | 183 |  |
| 171 |  | 184 |  |
| 172 |  | 185 |  |
| 173 |  | 186 |  |
| 174 |  | 187 |  |
| 188 |  | 200 |  |
| 189 |  | 201 |  |
| 190 |  | 202 |  |
| 191 |  | 203 |  |
| 192 |  | 204 |  |
| 193 |  | 205 |  |
| 194 |  | 206 |  |
| 195 |  | 207 |  |
| 196 |  | 208 |  |
| 197 |  | 209 |  |
| 198 |  | 210 |  |
| 199 |  | 211 |  |
| 212 |  | 226 |  |
| 213 |  | 227 |  |
| 214 |  | 228 |  |
| 215 |  | 229 |  |
| 216 |  | 230 |  |
| 217 |  | 231 |  |
| 218 |  | 232 |  |
| 219 |  | 233 |  |
| 220 |  | 234 |  |
| 221 |  | 235 |  |
| 222 |  | 236 |  |
| 223 |  | 237 |  |
| 224 |  | 238 |  |
| 225 |  | 239 |  |
| 240 |  | 253 |  |
| 241 |  | 254 |  |
| 242 |  | 255 |  |
| 243 |  | 256 |  |
| 244 |  | 257 |  |
| 245 |  | 258 |  |
| 246 |  | 259 |  |
| 247 |  | 260 |  |
| 248 |  | 261 |  |
| 249 |  | 262 |  |
| 250 |  | 263 |  |
| 251 |  | 264 |  |
| 252 |  | 265 |  |
|  |  |  |  |
| 266 |  | 279 |  |
| 267 |  | 280 |  |
| 268 |  | 281 |  |
| 269 |  | 282 |  |
| 270 |  | 283 |  |
| 271 |  | 284 |  |
| 272 |  | 285 |  |
| 273 |  | 286 |  |
| 274 |  | 287 |  |
| 275 |  | 288 |  |
| 276 |  | 289 |  |
| 277 |  | 290 |  |
| 278 |  | 291 |  |
| 292 |  | 305 |  |
| 293 |  | 306 |  |
| 294 |  | 307 |  |
| 295 |  | 308 |  |
| 296 |  | 309 |  |
| 297 |  | 310 |  |
| 298 |  | 311 |  |
| 299 |  | 312 |  |
| 300 |  | 313 |  |
| 301 |  | 314 |  |
| 302 |  | 315 |  |
| 303 |  | 316 |  |
| 304 |  | 317 |  |
| 318 |  |  |  |
| 319 |  |  |  |
| 320 |  |  |  |
| 321 |  |  |  |
| 322 |  |  |  |
| 323 |  |  |  |
| 324 |  |  |  |
| 325 |  |  |  |
| 326 |  |  |  |
| 327 |  |  |  |
| 328 |  |  |  |
