# Supplementary material for: An In Silico Approach for Modelling T-Helper Polarizing iNKT Cell Agonists
Source: PLoS One. 2014 Jan 31;9(1):e87000. doi: 10.1371/journal.pone.0087000 (PMC3909045; doi:10.1371/journal.pone.0087000)
Supplement: File S2 — References. (DOCX) [file pone.0087000.s002.docx]

# Supporting information S2

**REFERENCES**

1. Aspeslagh S, Li Y, Yu ED, Pauwels N, Trappeniers M, Girardi E, et al. Galactose-modified iNKT cell agonists stabilized by an induced fit of CD1d prevent tumour metastasis. The EMBO journal. 2011 Jun 1;30(11):2294-305.

2. Bricard G, Venkataswamy MM, Yu KO, Im JS, Ndonye RM, Howell AR, et al. Alpha-galactosylceramide analogs with weak agonist activity for human iNKT cells define new candidate anti-inflammatory agents. PloS one. 2010;5(12):e14374.

3. Brossay L, Naidenko O, Burdin N, Matsuda J, Sakai T, Kronenberg M. Cutting edge: Structural requirements for galactosylceramide recognition by CD1-restricted NK T cells. Journal of immunology. 1998 Nov 15;161(10):5124-8.

4. Chang DH, Deng H, Matthews P, Krasovsky J, Ragupathi G, Spisek R, et al. Inflammation-associated lysophospholipids as ligands for CD1d-restricted T cells in human cancer. Blood. 2008 Aug 15;112(4):1308-16.

5. Chang YJ, Huang JR, Tsai YC, Hung JT, Wu D, Fujio M, et al. Potent immune-modulating and anticancer effects of NKT cell stimulatory glycolipids. Proceedings of the National Academy of Sciences of the United States of America. 2007 Jun 19;104(25):10299-304.

6. Chen WL, Xia CF, Wang JH, Thapa P, Li YS, Nadas J, et al. Synthesis and structure activity relationship study of isoglobotrihexosylceramide analogues. J Org Chem. 2007 Dec 21;72(26):9914-23.

7. Fan G-T, Pan Y-s, Lu K-C, Cheng Y-P, Lin W-C, Lin S, et al. Synthesis of α-galactosyl ceramide and the related glycolipids for evaluation of their activities on mouse splenocytes. Tetrahedron. 2005;61(7):1855-62.

8. Fischer K, Scotet E, Niemeyer M, Koebernick H, Zerrahn J, Maillet S, et al. Mycobacterial phosphatidylinositol mannoside is a natural antigen for CD1d-restricted T cells. Proceedings of the National Academy of Sciences of the United States of America. 2004 Jul 20;101(29):10685-90.

9. Franchini L, Matto P, Ronchetti F, Panza L, Barbieri L, Costantino V, et al. Synthesis and evaluation of human T cell stimulating activity of an alpha-sulfatide analogue. Bioorganic & medicinal chemistry. 2007 Aug 15;15(16):5529-36.

10. Fuhshuku K, Hongo N, Tashiro T, Masuda Y, Nakagawa R, Seino K, et al. RCAI-8, 9, 18, 19, and 49-52, conformationally restricted analogues of KRN7000 with an azetidine or a pyrrolidine ring: Their synthesis and bioactivity for mouse natural killer T cells to produce cytokines. Bioorganic & medicinal chemistry. 2008 Jan 15;16(2):950-64.

11. Fujio M, Wu DG, Garcia-Navarro R, Ho DD, Tsuji M, Wong CH. Structure-based discovery of glycolipids for CD1d-mediated NKT cell activation: Tuning the adjuvant versus immunosuppression activity. Journal of the American Chemical Society. 2006 Jul 19;128(28):9022-3.

12. Goff RD, Gao Y, Mattner J, Zhou DP, Yin N, Cantu C, et al. Effects of lipid chain lengths in alpha-galactosylceramides on cytokine release by natural killer T cells. Journal of the American Chemical Society. 2004 Oct 27;126(42):13602-3.

13. Gumperz JE, Roy C, Makowska A, Lum D, Sugita M, Podrebarac T, et al. Murine CD1d-restricted T cell recognition of cellular lipids. Immunity. 2000 Feb;12(2):211-21.

14. Harrak Y, Barra CM, Delgado A, Castano AR, Llebaria A. Galacto-configured aminocyclitol phytoceramides are potent in vivo invariant natural killer T cell stimulators. Journal of the American Chemical Society. 2011 Aug 10;133(31):12079-84.

15. Hogan AE, O'Reilly V, Dunne MR, Dere RT, Zeng SG, O'Brien C, et al. Activation of human invariant natural killer T cells with a thioglycoside analogue of alpha-galactosylceramide. Clinical immunology. 2011 Aug;140(2):196-207.

16. Hunault J, Diswall M, Frison JC, Blot V, Rocher J, Marionneau-Lambot S, et al. 3-fluoro- and 3,3-difluoro-3,4-dideoxy-KRN7000 analogues as new potent immunostimulator agents: total synthesis and biological evaluation in human invariant natural killer T cells and mice. Journal of medicinal chemistry. 2012 Feb 9;55(3):1227-41.

17. Im JS, Arora P, Bricard G, Molano A, Venkataswamy MM, Baine I, et al. Kinetics and cellular site of glycolipid loading control the outcome of natural killer T cell activation. Immunity. 2009 Jun 19;30(6):888-98.

18. Jervis PJ, Moulis M, Jukes JP, Ghadbane H, Cox LR, Cerundolo V, et al. Towards multivalent CD1d ligands: synthesis and biological activity of homodimeric alpha-galactosyl ceramide analogues. Carbohydrate research. 2012 Jul 15;356:152-62.

19. Kerzerho J, Yu ED, Barra CM, Alari-Pahissa E, Girardi E, Harrak Y, et al. Structural and functional characterization of a novel nonglycosidic type I NKT agonist with immunomodulatory properties. Journal of immunology. 2012 Mar 1;188(5):2254-65.

20. Kinjo Y, Illarionov P, Vela JL, Pei B, Girardi E, Li X, et al. Invariant natural killer T cells recognize glycolipids from pathogenic Gram-positive bacteria. Nature immunology. 2011 Oct;12(10):966-74.

21. Kinjo Y, Illarionov P, Vela JL, Pei B, Girardi E, Li X, et al. Invariant natural killer T cells recognize glycolipids from pathogenic Gram-positive bacteria. Nature immunology. 2011 Oct;12(10):966-74.

22. Kinjo Y, Pei B, Bufali S, Raju R, Richardson SK, Imamura M, et al. Natural Sphingomonas glycolipids vary greatly in their ability to activate natural killer T cells. Chemistry & biology. 2008 Jul 21;15(7):654-64.

23. Kinjo Y, Tupin E, Wu D, Fujio M, Garcia-Navarro R, Benhnia MR, et al. Natural killer T cells recognize diacylglycerol antigens from pathogenic bacteria. Nature immunology. 2006 Sep;7(9):978-86.

24. Lee T, Cho M, Ko SY, Youn HJ, Baek DJ, Cho WJ, et al. Synthesis and evaluation of 1,2,3-triazole containing analogues of the immunostimulant alpha-GalCer. Journal of medicinal chemistry. 2007 Feb 8;50(3):585-9.

25. Li X, Chen G, Garcia-Navarro R, Franck RW, Tsuji M. Identification of C-glycoside analogues that display a potent biological activity against murine and human invariant natural killer T cells. Immunology. 2009;127(2):216-25.

26. Li XM, Fujio M, Imamura M, Wu D, Vasan S, Wong CH, et al. Design of a potent CD1d-binding NKT cell ligand as a vaccine adjuvant. Proceedings of the National Academy of Sciences of the United States of America. 2010 Jul 20;107(29):13010-5.

27. Liang PH, Imamura M, Li XM, Wu D, Fujio M, Guy RT, et al. Quantitative microarray analysis of intact glycolipid - CD1d interaction and correlation with cell-based cytokine production. Journal of the American Chemical Society. 2008 Sep 17;130(37):12348-54.

28. Liu Y, Goff RD, Zhou D, Mattner J, Sullivan BA, Khurana A, et al. A modified alpha-galactosyl ceramide for staining and stimulating natural killer T cells. Journal of immunological methods. 2006 May 30;312(1-2):34-9.

29. Long XT, Deng S, Mattner JC, Zang Z, Zhou D, McNary N, et al. Synthesis and evaluation of stimulatory properties of Sphingomonadaceae glycolipids. Nat Chem Biol. 2007 Sep;3(9):559-64.

30. Lotter H, Gonzalez-Roldan N, Lindner B, Winau F, Isibasi A, Moreno-Lafont M, et al. Natural Killer T Cells Activated by a Lipopeptidophosphoglycan from Entamoeba histolytica Are Critically Important To Control Amebic Liver Abscess. Plos Pathog. 2009 May;5(5).

31. Mattner J, DeBord KL, Ismail N, Goff RD, Cantu C, Zhou DP, et al. Exogenous and endogenous glycolipid antigens activate NKT cells during microbial infections. Nature. 2005 Mar 24;434(7032):525-9.

32. McCarthy C, Shepherd D, Fleire S, Stronge VS, Koch M, Illarionov PA, et al. The length of lipids bound to human CD1d molecules modulates the affinity of NKT cell TCR and the threshold of NKT cell activation. The Journal of experimental medicine. 2007 May 14;204(5):1131-44.

33. Miyamoto K, Miyake S, Yamamura T. A synthetic glycolipid prevents autoimmune encephalomyelitis by inducing T(H)2 bias of natural killer T cells. Nature. 2001 Oct 4;413(6855):531-4.

34. Ndonye RM, Izmirian DP, Dunn MF, Yu KOA, Porcelli SA, Khurana A, et al. Synthesis and evaluation of sphinganine analogues of KRN7000 and OCH. J Org Chem. 2005 Dec 9;70(25):10260-70.

35. O'Konek JJ, Illarionov P, Khursigara DS, Ambrosino E, Izhak L, Castillo BF, 2nd, et al. Mouse and human iNKT cell agonist beta-mannosylceramide reveals a distinct mechanism of tumor immunity. The Journal of clinical investigation. 2011 Feb;121(2):683-94.

36. Parekh VV, Singh AK, Wilson MT, Olivares-Villagomez D, Bezbradica JS, Inazawa H, et al. Quantitative and qualitative differences in the in vivo response of NKT cells to distinct alpha- and beta-anomeric glycolipids. Journal of immunology. 2004 Sep 15;173(6):3693-706.

37. Park JJ, Lee JH, Seo KC, Bricard G, Venkataswamy MM, Porcelli SA, et al. Syntheses and biological activities of KRN7000 analogues having aromatic residues in the acyl and backbone chains with varying stereochemistry. Bioorganic & medicinal chemistry letters. 2010 Feb 1;20(3):814-8.

38. Prigozy TI, Naidenko O, Qasba P, Elewaut D, Brossay L, Khurana A, et al. Glycolipid antigen processing for presentation by CD1d molecules. Science. 2001 Jan 26;291(5504):664-7.

39. Raju R, Castillo BF, Richardson SK, Thakur M, Severins R, Kronenberg M, et al. Synthesis and evaluation of 3''- and 4''-deoxy and -fluoro analogs of the immunostimulatory glycolipid, KRN7000. Bioorganic & medicinal chemistry letters. 2009 Aug 1;19(15):4122-5.

40. Reddy BG, Silk JD, Salio M, Balamurugan R, Shepherd D, Ritter G, et al. Nonglycosidic Agonists of Invariant NKT Cells for Use as Vaccine Adjuvants. Chemmedchem. 2009 Feb;4(2):171-5.

41. Shiozaki M, Tashiro T, Koshino H, Nakagawa R, Inoue S, Shigeura T, et al. Synthesis and biological activity of ester and ether analogues of alpha-galactosylceramide (KRN7000). Carbohydrate research. 2010 Aug 16;345(12):1663-84.

42. Sidobre S, Hammond KJ, Benazet-Sidobre L, Maltsev SD, Richardson SK, Ndonye RM, et al. The T cell antigen receptor expressed by Valpha14i NKT cells has a unique mode of glycosphingolipid antigen recognition. Proceedings of the National Academy of Sciences of the United States of America. 2004 Aug 17;101(33):12254-9.

43. Silk JD, Salio M, Reddy BG, Shepherd D, Gileadi U, Brown J, et al. Nonglycosidic CD1d lipid ligands activate human and murine invariant NKT cells. Journal of immunology. 2008 May 15;180(10):6452-6.

44. Tashiro T, Nakagawa R, Inoue S, Shiozaki M, Watarai H, Taniguchi M, et al. RCAI-61, the 6′-O-methylated analog of KRN7000: its synthesis and potent bioactivity for mouse lymphocytes to produce interferon-γ in vivo. Tetrahedron Letters. 2008;49(48):6827-30.

45. Trappeniers M, Chofor R, Aspeslagh S, Li Y, Linclau B, Zajonc DM, et al. Synthesis and evaluation of amino-modified alpha-GalCer analogues. Organic letters. 2010 Jul 2;12(13):2928-31.

46. Trappeniers M, Van Beneden K, Decruy T, Hillaert U, Linclau B, Elewaut D, et al. 6 '-Derivatised alpha-GalCer Analogues Capable of Inducing Strong CD1d-Mediated Th1-Biased NKT Cell Responses in Mice. Journal of the American Chemical Society. 2008 Dec 10;130(49):16468-+.

47. Wojno J, Jukes JP, Ghadbane H, Shepherd D, Besra GS, Cerundolo V, et al. Amide analogues of CD1d agonists modulate iNKT-cell-mediated cytokine production. ACS chemical biology. 2012 May 18;7(5):847-55.

48. Wu D, Xing GW, Poles MA, Horowitz A, Kinjo Y, Sullivan B, et al. Bacterial glycolipids and analogs as antigens for CD1d-restricted NKT cells. Proceedings of the National Academy of Sciences of the United States of America. 2005 Feb 1;102(5):1351-6.

49. Wu D, Zajonc DM, Fujio M, Sullivan BA, Kinjo Y, Kronenberg M, et al. Design of natural killer T cell activators: structure and function of a microbial glycosphingolipid bound to mouse CD1d. Proceedings of the National Academy of Sciences of the United States of America. 2006 Mar 14;103(11):3972-7.

50. Wu TN, Lin KH, Chang YJ, Huang JR, Cheng JY, Yu AL, et al. Avidity of CD1d-ligand-receptor ternary complex contributes to T-helper 1 (Th1) polarization and anticancer efficacy. Proceedings of the National Academy of Sciences of the United States of America. 2011 Oct 18;108(42):17275-80.

51. Yin N, Long X, Goff RD, Zhou D, Cantu C, 3rd, Mattner J, et al. Alpha anomers of iGb3 and Gb3 stimulate cytokine production by natural killer T cells. ACS chemical biology. 2009 Mar 20;4(3):199-208.

52. Yoshiga Y, Goto D, Segawa S, Horikoshi M, Hayashi T, Matsumoto I, et al. Activation of natural killer T cells by alpha-carba-GalCer (RCAI-56), a novel synthetic glycolipid ligand, suppresses murine collagen-induced arthritis. Clinical and experimental immunology. 2011 May;164(2):236-47.

53. Yu KO, Im JS, Molano A, Dutronc Y, Illarionov PA, Forestier C, et al. Modulation of CD1d-restricted NKT cell responses by using N-acyl variants of alpha-galactosylceramides. Proceedings of the National Academy of Sciences of the United States of America. 2005 Mar 1;102(9):3383-8.

54. Zajonc DM, Cantu C, 3rd, Mattner J, Zhou D, Savage PB, Bendelac A, et al. Structure and function of a potent agonist for the semi-invariant natural killer T cell receptor. Nature immunology. 2005 Aug;6(8):810-8.

55. Zhang W, Xia C, Nadas J, Chen W, Gu L, Wang PG. Introduction of aromatic group on 4'-OH of alpha-GalCer manipulated NKT cell cytokine production. Bioorganic & medicinal chemistry. 2011 Apr 15;19(8):2767-76.

56. Zhou D, Mattner J, Cantu C, 3rd, Schrantz N, Yin N, Gao Y, et al. Lysosomal glycosphingolipid recognition by NKT cells. Science. 2004 Dec 3;306(5702):1786-9.

57. Zhou XT, Forestier C, Goff RD, Li CH, Teyton L, Bendelac A, et al. Synthesis and NKT cell stimulating properties of fluorophore- and biotin-appended 6 ''-amino-6 ''-deoxy-galactosylceramides. Organic letters. 2002 Apr 18;4(8):1267-70.
