# Supplementary material for: An In Silico Approach for Modelling T-Helper Polarizing iNKT Cell Agonists
Source: PLoS One. 2014 Jan 31;9(1):e87000. doi: 10.1371/journal.pone.0087000 (PMC3909045; doi:10.1371/journal.pone.0087000)
Supplement: File S3 — Clustering methods. (DOCX) [file pone.0087000.s003.docx]

# Supporting information S3

**CLUSTERING METHODS**

In order to calculate the chemical descriptors of the 333 glycolipids, the three-dimensional structure was optimized using Hyperchem 8.0.8 (Hypercube, Gainesville, FL, USA). Therefore, the molecular mechanics force field method was applied, using the Polak-Ribière conjugate gradient algorithm with a root mean square gradient of 0.1 kcal/(Å×mol) as stop criterion. Next, 3228 descriptors were calculated, using the Dragon 5.5 (Talete, Milan, Italy) and Hyperchem 8.0.8 software programs: using Hyperchem, the surface area (Å^2^), volume (Å^3^), logP and refractivity (Å^3^) of all molecules was calculated using the 3D-optimized .mol-files, and these descriptors were added to the Dragon-obtained descriptor values. After removal of the constant descriptors, which are thus not discriminative, a final dataset of 1656 descriptors was retained. As each descriptor differed in the scales in which their values lie, normalization was performed by Unit Variance (UV) scaling and mean centering [12]. This resulted in a 333 x 1656 matrix of z-scaled descriptor values: $z=\frac{x- \bar{x}}{SD}$, where SD is the standard deviation and $\bar{x}$ the mean of each variable.

The multivariate data-analysis on this resulting data-matrix was performed using Principal Component Analysis (PCA) and Hierarchical Cluster Analysis (HCA) with SIMCA-P+ 12.0 (Umetrics, Sweden) and SPSS Statistics 20.0 (Illinois, USA) software programs, respectively. The dendrogram of the HCA analysis was obtained using average-linkage clustering with the Euclidean distance as the dissimilarity criterion [13].
