# Supplementary material for: An In Silico Approach for Modelling T-Helper Polarizing iNKT Cell Agonists
Source: PLoS One. 2014 Jan 31;9(1):e87000. doi: 10.1371/journal.pone.0087000 (PMC3909045; doi:10.1371/journal.pone.0087000)
Supplement: File S4 — Derringer transformation. (DOCX) [file pone.0087000.s004.docx]

# Supporting information S4

**DERRINGER TRANSFORMATION**

The biological responses were transformed into a dimensionless desirability (d) scale via the following linear desirability functions:

$d\left( Y \right)= \frac{0.9-0.1}{Y_{max}- Y_{min}} \times\left( Y_{i}-Y_{min} \right)+ 0.1$

or $d\left( Y \right)= \frac{0.1-0.9}{Y_{max}- Y_{min}} \times\left( Y_{i}-Y_{min} \right)+ 0.9$

for parameters to be maximized or minimized respectively. In the equations above, Y_i_ is the experimental value for the respective response, whereas Y_min_ and Y_max_ are the minimum and maximum response values found, respectively. Y_min_ was arbitrarily set at the reporting threshold of 0.01 for every response; Y_max_ is the maximal value found for each methodology in our dataset and their values are reported in *Supporting information S5*. Compounds with a high Th1 desirability should have high IFN-γ values, combined with low IL-4 responses. This contrasts the Th2 desirability: high IL-4 responses combined with low IFN-γ values are required. After this linear d-transformation of each of the responses, all values range from 0.1 (undesirable) to 0.9 (most desirable). These standardized d-values were combined to calculate a global D-value:

$$D= \sqrt[n]{\prod_{i=1}^{n} d_{i}^{Pi}}$$

The compound with the highest D-value expresses the best combination of the different desired responses. In this equation, p_i_ is the relative importance given to the respective response. Here, we weighted the responses equally, so p_i_ = 1 for each of the 20 responses.
