# Supplementary material for: An In Silico Approach for Modelling T-Helper Polarizing iNKT Cell Agonists
Source: PLoS One. 2014 Jan 31;9(1):e87000. doi: 10.1371/journal.pone.0087000 (PMC3909045; doi:10.1371/journal.pone.0087000)
Supplement: File S5 — Maximally found response values Ymax. (DOCX) [file pone.0087000.s005.docx]

# Supporting information S5

MAXIMALLY FOUND RESPONSE VALUES Ymax.

| **Test-system** | **Marker** | **Y_max_** |
| --- | --- | --- |
| *Mice/in-vivo* | IL-2 | 1.8 |
|  | IFN- γ | 2.7 |
|  | IL-4 | 3.4 |
|  | IL-13 | Not available |
| *Mice/in-vitro/cell-cell* | IL-2 | 2.5 |
|  | IFN- γ | 3.2 |
|  | IL-4 | 2.9 |
|  | IL-13 | 1.6 |
| *Mice/in-vitro/cell-plate* | IL-2 | 26 |
|  | IFN- γ | Not available |
|  | IL-4 | Not available |
|  | IL-13 | Not available |
| *Human/in-vitro/cell-cell* | IL-2 | 2.7 |
|  | IFN- γ | 2.5 |
|  | IL-4 | 2.5 |
|  | IL-13 | 1.9 |
| *Human/in-vitro/cell-plate* | IL-2 | 1.0 |
|  | IFN- γ | 1.0 |
|  | IL-4 | Not available |
|  | IL-13 | Not available |
