# Supplementary material for: An In Silico Approach for Modelling T-Helper Polarizing iNKT Cell Agonists
Source: PLoS One. 2014 Jan 31;9(1):e87000. doi: 10.1371/journal.pone.0087000 (PMC3909045; doi:10.1371/journal.pone.0087000)
Supplement: File S6 — PCA model description. (DOCX) [file pone.0087000.s006.docx]

# Supporting information S6

**PCA MODEL DESCRIPTION**

Our calculated PCA model contains eight principal components (PCs), with 48.5% of the variability explained by the first two principal components. The eigenvalues of the covariance matrix and the total variance explained (R^2^) are given.

| Principal Component | Eigenvalue | Cumulative R^2^ |
| --- | --- | --- |
| 1 | 117 | 0.353 |
| 2 | 44.1 | 0.485 |
| 3 | 19.2 | 0.543 |
| 4 | 16.0 | 0.591 |
| 5 | 13.2 | 0.630 |
| 6 | 9.49 | 0.659 |
| 7 | 8.52 | 0.684 |
| 8 | 8.27 | 0.709 |
