# Supplementary figures and images for: An In Silico Approach for Modelling T-Helper Polarizing iNKT Cell Agonists
Source: PLoS One. 2014 Jan 31;9(1):e87000. doi: 10.1371/journal.pone.0087000 (PMC3909045; doi:10.1371/journal.pone.0087000)

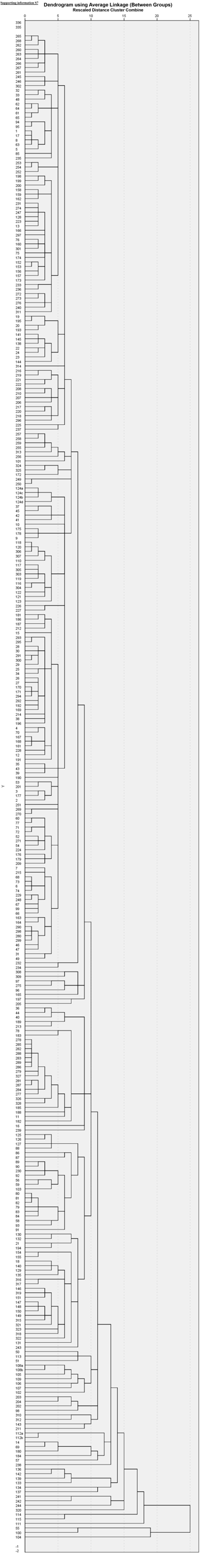

Supplement: File S7 — Dendrogram using Average Linkage. (TIF) [file pone.0087000.s007.tif]

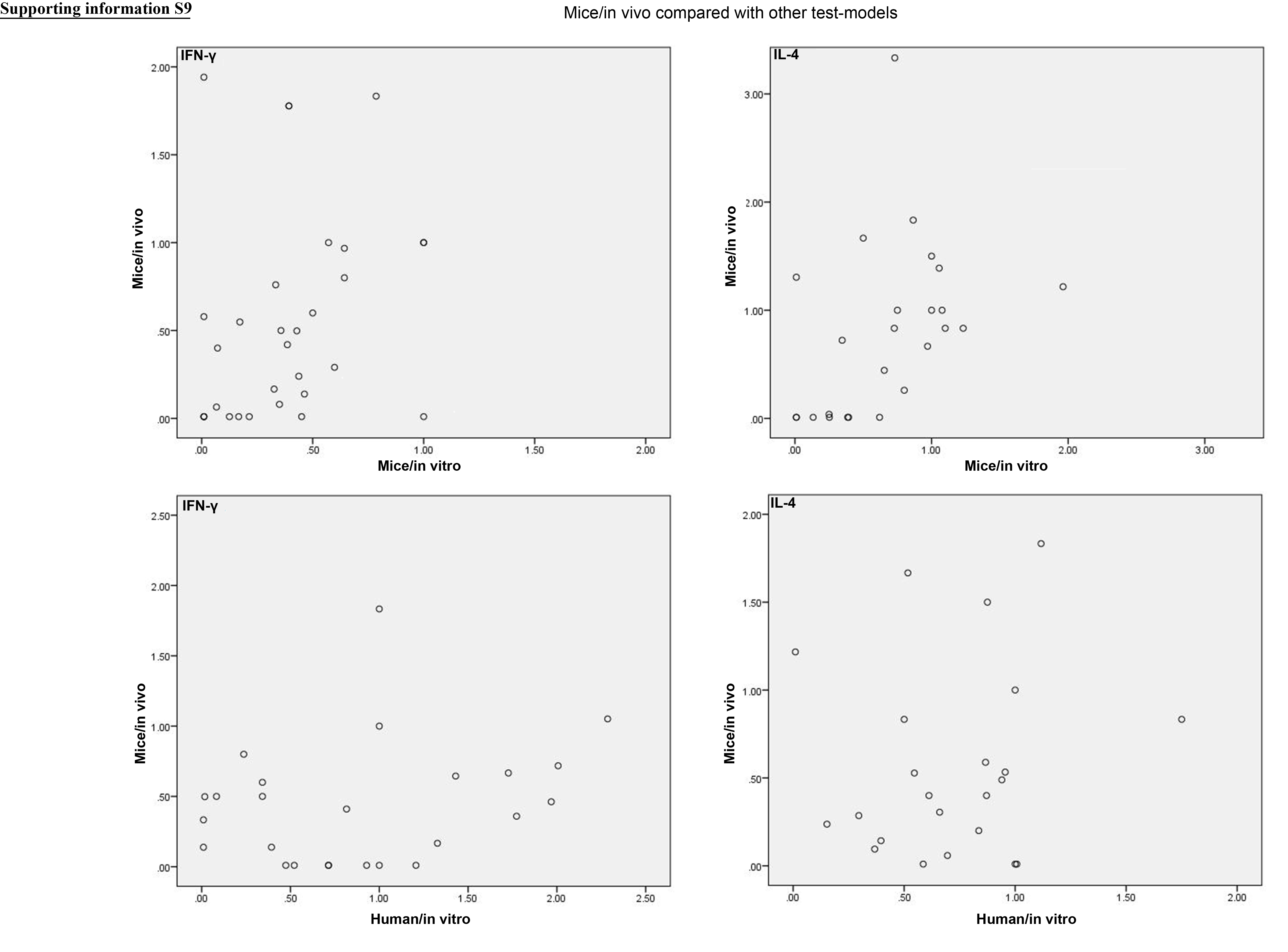

Supplement: File S9 — Mice/in vivo compared with other test-models. (TIF) [file pone.0087000.s009.tif]
