# Supplementary material for: An In Silico Approach for Modelling T-Helper Polarizing iNKT Cell Agonists
Source: PLoS One. 2014 Jan 31;9(1):e87000. doi: 10.1371/journal.pone.0087000 (PMC3909045; doi:10.1371/journal.pone.0087000)
Supplement: File S8 — Results of HCA-clustering. (DOCX) [file pone.0087000.s008.docx]

# Supporting information S8

**RESULTS OF HCA-CLUSTERING**

Another chemically distinct group are α-GalCer analogs lacking the characteristic carbohydrate moiety. In our analysis, they are organized in different subclusters. First, the substitution of the sugar group by a cyclitol results in different chemical properties (*e.g.* **50**, **51**, **113**-**115**, **245**, **246**, **302**) and second, the threitolceramide-derived molecules (*e.g.* **255-268**) are also separately clustered. Next, the sugar moiety can be modified by the addition of different functional groups as well, *e.g.* the 3,4-dichlorophenyl (**133**, **136**, **139** and **142**) and 4-chlorophenyl (**21**, **130**, **132** and **194**) modifications, each divided into a separate cluster of the dendrogram. The addition of a ring structure on the sugar molecule is also a common feature: phenyl (**19**, **20**, **22**, **23**, **24**, **138**, **141**, **144**, **145**, **193** and **195**), naphthalene (**18**, **129**, **135** and **140**), 1,2,3-triazole, with or without a phenyl ring structure (**146-151** and **315-323**) and pyridine (**131**). Interestingly, there is no chemical difference seen based on the type of linker between the sugar and the 6-OH alteration: carbamate, ureum or amide linkers are clustered in the same group, independent of the number of linker atoms between the sugar and the modification. So, one cluster can contain molecules with an ureum linker (**19**) as well as an amide linker of variable length (**138**, **141** and **145**). Consequently, the type of modification (*e.g.* phenyl, 3,4-dichlorophenyl) apparently influences the clustering the most. S-containing molecules have various chemical characteristics as well, due to the presence of *e.g.* a naphthalene-1-sulfonamide moiety (**241** and **242**) and sulphate or thiol functional groups (**98**, **202**, **203**, **204** and **211**). In addition, the presence of a fluor atom, directly on the sugar molecule (**96**, **97**, **205** and **275**) or on a phenyl ring as trifluoromethyl, in combination with a chloride-substitution (**134** and **137**), is also characteristic for a separate clustering.

Beside galactose modifications, several groups have provided analogs with ceramide modifications as well. A first group consists of glycolipids with shortened fatty acid chains (**14**, **57**, **69** and **180**). A second group is characterized by the introduction of a four-membered (**216**, **219**, **221** and **222**) or five-membered (**217**, **218**, **220** and **296**) ring structure between the acyl chains and sugar moiety. A third alteration possibility includes the incorporation of a fluor atom into the ceramide group: a trifluoromethyl group on a phenyl ring (**36**, **40**, **44**, **189** and **213**), a difluorophenyl functional group (**16**) or one fluor atom, whether or not attached to a phenyl ring (*e.g.* **35**, **39**, **43**, **308**, **309**, **326** and **328**) can be incorporated. Next to the cyclic fluor-containing modifications, phenyl (*e.g.* **25-33**, **169-171**, **186**, **291-295** and **300**), biphenyl (**37**, **41**, **45**), methoxyphenyl (**34**, **38**, **42**, **181** and **187**), propylphenyl (**214**), methylphenyl (**15**), chlorobenzene (**182**), bromobenzene (**238**), tricyclodecane (**239**), thiophene (**184**), piperidine (**191**), 1,2,3-triazole (**249-254**), pyridine (**183**) and naphthalene (**185**) rings are also present on the N-acyl group. However, the (bi)phenyl and methoxyphenyl containing molecules are not clustered separately, indicating similar chemical properties for these compounds. Moreover, a phenyl ring can be present on both the N-acyl and phytosphingosine-chain (**277-279**, **281-289** and **327**); these molecules are again grouped in a separate branch of the dendrogram. If the phenyl (**46**, **47**, **163**, **164**, **280**, **290**, **298** and **299**), phtalamide (**154** and **155**) or cyclopropane ring (**124a**, **124b**, **124c** and **124d**) is only attached to the phytosphingosine-chain, this also results in an individual clustering. Finally, the triple bond containing alkyl chains (**232** and **234**) are clustered in one group, while the alpha-galactosylceramide molecules with a double bond are not.
