# Supplementary material for: An In Silico Approach for Modelling T-Helper Polarizing iNKT Cell Agonists
Source: PLoS One. 2014 Jan 31;9(1):e87000. doi: 10.1371/journal.pone.0087000 (PMC3909045; doi:10.1371/journal.pone.0087000)
Supplement: File S10 — MLR and PLS models. (DOCX) [file pone.0087000.s010.docx]

**Supporting information S10**

**MLR AND PLS MODELS**

**Table S10A: Stepwise MLR model: Th1 *in vivo***

| **Th1 *in vivo*** | | | |
| --- | --- | --- | --- |
| **Descriptor** | **Unstandardized Coefficients** | **Standardized Coefficients** | **Adjusted R^2^** |
| Constant | .007 | - |  |
| nCt | .654 | .516 |  |
| B05[N-O] | .218 | .498 |  |
| C-041 | .150 | .389 |  |
| Mor04e | .005 | .458 |  |
| F08[N-O] | -.036 | -.250 |  |
| Mor22m | .073 | .250 | **0.685** |
| Mor20u | -.041 | -.245 |  |
| F-084 | .068 | .138 |  |
| B06[O-S] | .125 | .170 |  |
| nROCON | -.137 | -.152 |  |
| nArOR | .130 | .145 |  |
| Mor02m | .002 | .155 |  |
| Mor26m | .064 | .133 |  |

**Table S10B: Stepwise MLR model: Th1 *in vitro***

| **Th1 *in vitro*** | | | |
| --- | --- | --- | --- |
| **Descriptor** | **Unstandardized Coefficients** | **Standardized Coefficients** | **Adjusted R^2^** |
| Constant | -1.289 | - |  |
| nCb- | .063 | .507 |  |
| G2e | -2.556 | -.292 |  |
| nRNH2 | -.122 | -.214 |  |
| R3m+ | -3.959 | -.420 |  |
| Mor31m | -.081 | -.193 |  |
| BEHe3 | 1.561 | .319 |  |
| MATS8m | -.465 | -.283 |  |
| GATS4v | -.876 | -.344 |  |
| nCONN | .226 | .164 |  |
| R4m | 1.193 | .567 |  |
| piPC07 | -.269 | -1.011 |  |
| HATS8e | -1.552 | -.432 |  |
| P2u | -.346 | -.246 | **0.825** |
| EEig07r | -.610 | -.442 |  |
| MSD | -2.031 | -.394 |  |
| nCconj | .022 | .103 |  |
| Mor20u | .031 | .183 |  |
| EEig04x | .156 | .240 |  |
| Mor26m | .072 | .218 |  |
| SPAM | .945 | .276 |  |
| Mor16v | .340 | 1.034 |  |
| RDF080e | .001 | .219 |  |
| Mor16p | -.255 | -.863 |  |
| B10[O-F] | -.134 | -.137 |  |
| Mor28e | .026 | .109 |  |
| B09[C-F] | .052 | .116 |  |
| G1m | -2.025 | -.103 |  |

**Table S10C: Stepwise MLR model: Th2 *in vivo***

| **Th2 *in vivo*** | | | |
| --- | --- | --- | --- |
| **Descriptor** | **Unstandardized Coefficients** | **Standardized Coefficients** | **Adjusted R^2^** |
| Constant | .121 | - |  |
| nR=Cs | .016 | .242 |  |
| H-048 | .116 | .555 |  |
| R8e+ | 11.657 | .384 |  |
| R3e+ | -9.130 | -.672 |  |
| B09[N-O] | -.069 | -.369 |  |
| C-034 | .227 | .457 |  |
| G(N..S) | -.034 | -.345 |  |
| RDF155u | .004 | 1.521 |  |
| W3D | -1.507E-006 | -1.002 |  |
| F07[O-O] | .051 | .595 |  |
| L/Bw | .002 | .319 |  |
| E3p | -.554 | -.302 |  |
| Mor20u | .076 | .583 |  |
| Mor31u | .062 | .810 |  |
| E1e | -.675 | -.258 |  |
| Mor13e | .022 | .283 | **0.862** |
| RDF150m | -.008 | -.407 |  |
| RDF020m | .121 | .455 |  |
| RDF070m | -.002 | -.225 |  |
| RDF120u | .002 | .583 |  |
| Mor28u | .043 | .213 |  |
| ECC | .000 | -1.557 |  |
| Dm | .628 | .380 |  |
| R6m+ | -3.497 | -.163 |  |
| B06[N-N] | -.116 | -.119 |  |
| Mor29v | .076 | .333 |  |
| G2v | 1.324 | .137 |  |
| C-041 | -.061 | -.205 |  |
| RDF155p | -.007 | -.880 |  |
| RDF140m | .003 | .259 |  |
| MATS8v | .249 | .153 |  |
| R4e+ | 2.118 | .141 |  |
| G2u | -.673 | -.139 |  |

**Table S10D: Stepwise MLR model: Th2 *in vitro***

| **Th2 *in vitro*** | | | |
| --- | --- | --- | --- |
| **Descriptor** | **Unstandardized Coefficients** | **Standardized Coefficients** | **Adjusted R^2^** |
| Constant | 3,539 | - |  |
| C-026 | -,036 | -,159 |  |
| B08[N-O] | ,154 | ,383 |  |
| G1u | -3,333 | -,306 |  |
| Mor28u | ,045 | ,239 |  |
| Mp | 9,980 | ,820 | **0.401** |
| BEHp1 | -,594 | -,460 |  |
| R3m+ | -2,523 | -,333 |  |
| BEHp3 | -1,771 | -,329 |  |
| nHBonds | ,019 | ,207 |  |
| C-033 | ,065 | ,151 |  |

**Table S10E: PLS model with cross-validation**

| **Functionality** | **Principal Components** | **Cumulative R^2^** | **Cumulative Q^2^** |
| --- | --- | --- | --- |
| Th1  *mice/in-vivo* | 7 | 0.941 | 0.742 |
| Th2  *mice/in-vivo* | 8 | 0.962 | 0.783 |
| Th1  *human/in-vitro*  *mice/in-vitro* | 4 | 0.722 | 0.512 |
| Th2  *human/in-vitro*  *mice/in-vitro* | 3 | 0.640 | 0.512 |
