# Supplementary material for: An In Silico Approach for Modelling T-Helper Polarizing iNKT Cell Agonists
Source: PLoS One. 2014 Jan 31;9(1):e87000. doi: 10.1371/journal.pone.0087000 (PMC3909045; doi:10.1371/journal.pone.0087000)
Supplement: File S11 — Meaning of described chemical descriptors. (DOCX) [file pone.0087000.s011.docx]

**Supporting information S11**

**MEANING OF DESCRIBED CHEMICAL DESCRIPTORS**

| **Name descriptor** | **Meaning** |
| --- | --- |
| nROCON | Number of (thio-) carbamates (aliphatic); R represents any group linked through carbon [1].  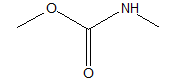 |
| C-041 | C-041 is an atom-centered fragment descriptor that counts the number of occurrences of C-atoms connected as X-C(=X)-X in the glycolipid molecule, where X represents any electronegative atom (O, N, S, P, Se, halogens) and = represents a double bond [2-4]. It thus corresponds to a certain chemical substructure present in the compound and can be considered as a pharmacophore descriptor.  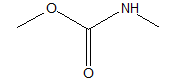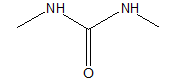 |
| Morxxe/m | 3D-MoRSE descriptors (signal xx, weighted by atomic Sanderson electronegativities (e) or atomic masses (m)) are 3-dimensional molecular descriptors representing the scattered electron intensity from a molecular beam. The intensity of scattered radiation is calculated from the 3D-atomic coordinates of a molecule for 32 different scattering angles. Morse descriptors contain information about atom distribution and the amount of branching within a molecule [5,6]. As the glycolipids mostly contain only C, N, O and F atoms, the electronegativities can be linked to the atomic masses (C < N < O < F). While the exact meaning remains still unclear, *i.a.* due to its sinus function [7,8], it has been demonstrated that Mor descriptors capture minimum variations in 3D-structural features based on interatomic distances, as calculated in vacuo. |
| F-084 | F attached to C^1^(sp2): *e.g.* F-atom attached to a benzene molecule [2-4]  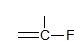 |
| Bx[C-F] | Presence/absence of C - F at topological distance x (01-10); presence = 1, absence = 0, topological distance = the number of bonds that separate the C and F atoms [9,10]. |
| nCb- | Number of substituted benzene C(sp2) [1]:  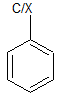 |
| nCONN | Number of urea (-thio) derivatives [1]:  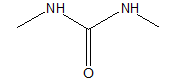 |
| H-048 | 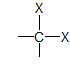H attached to C^2^(sp3)/C^1^(sp2)/C^0^(sp); the superscript represents the formal oxidation number. The formal oxidation number of a carbon atom equals the sum of the conventional bond orders with electronegative atoms [2-4].  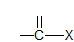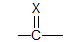  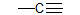 |
| Ui | Unsaturation index: gives an idea about the degree of unsaturation in the molecule [11] |
| nBM | Number of multiple bonds [1] |
| nCconj | Number of non-aromatic conjugated C(sp2) [1]:  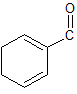 |
| Mp | Mean atomic polarizability (scaled on Carbon atom) [5] |
| nHBonds | Number of intramolecular H-bonds (with N,O,F):  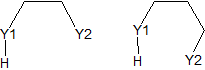  Y1 = B, N, O, P, S, aliphatic group, Y2 = N, O, F. The geometric distance between H and Y2 must be in the range 1 – 2.7 Å [1]. |
| nOHp | Number of primary alcohols [1]:  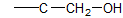 |
| ECC | Eccentricity: this descriptor gives information about the compactness of the structure [5]. |
| W3D | 3D-Wiener index: counts the number of bonds between pairs of atoms and sums the distances between all pairs. This descriptor gives thus an idea about the degree of branching of the molecule [12,13]. |
| Bx[N-N] | Presence/absence of N - N at topological distance x (01-10); presence = 1, absence = 0, topological distance = the number of bonds that separate the N atoms [9,10]. |
| Fx[N-N] | Frequency of N - N at topological distance x (01-10); topological distance = the number of bonds that separate the N atoms [9,10]. |
| nTriazoles | Number of Triazoles [1]:  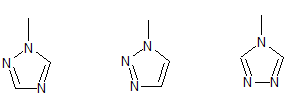 |
| N-073 | Ar2NH / Ar3N / Ar2N-Al / R..N..R; Ar represents an aromatic group, .. represents aromatic single bonds as the C-N bond in pyrrole. This descriptor gives information correlated to a pyrrole-type structure [2-4]. |
| nR=Cs | Number of aliphatic secondary C(sp2): this descriptor reflects the degree of unsaturation of the glycolipid [1].  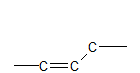 |
| C-034 | C-034 is an atom-centered fragment descriptor that counts the number of occurrences of C-atoms connected as R--CR..X, where -- represents an aromatic bond as in benzene or delocalized bonds such as the N-O bond in a nitro group, .. represents aromatic single bonds as the C-N bond in pyrrole, X symbolizes any electronegative atom (O, N, S, P, Se, halogens) and R corresponds to any group linked through carbon [2-4].  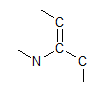 |

References:

[1] http://michem.disat.unimib.it/chm/Help/edragon/FunctionalGroupCounts11.html (13 November 2013)

[2] Viswanadhan, V. N.; Ghose, A. K.; Revankar, G. R.; Robins, R. K., *J. Chem. Inf. Comput. Sci.*, 1989, *29*, 163-172.

[3] Ghose, A.K.; Viswanadhan, V.N.; Wendoloski, J.J., *J. Phys. Chem. A*, 1998, *102*, 3762-3772.

[4] http://www.talete.mi.it/help/dproperties_help/index.html?atom_centred_fragments.htm (13 November 2013)

[5] Todeschini, R.; Consonni, V., Handbook of chemoinformatics: from data to knowledge in 4 volumes, 2008.

[6] Schuur, J.; Gasteiger, J., *Anal. Chem.,* 1997, *69*, 2398-2405.

[7] Saiz-Urra, L.; Gonzalez, M.P.; Teijeira, M., *Bioorg. Med. Chem.*, 2006, *14*, 7347-7358.

[8] Palomba, D.; Martinez, M.J.; Ponzoni, I.; Diaz, M.F.; Vasquez, G.E.; Soto, A.J., *Molecules*, 2012, *17*, 14937-14953.

[9] Varnek, A.; Tropsha, A.; Cheminformatics approaches to virtual screening, 2008.

[10] Balaban, A.T.; Bonchev, D.; Seitz, W.A., *J. Mol. Struct.*, 1993, *280*, 253-260.

[11] http://www.talete.mi.it/help/dproperties_help/index.html?molecular_properties.htm (13 November 2013)

[12] Mekenyan, O.; Peitchev, D.; Bonchev, D.; Trinajstic, N.; Bangov, I.P., *Arzneim. Forsch*., 1986, *36*, 176-183.

[13] Bogdanov, B.; Nikolic, S.; Trinajstic, N., *J. Math. Chem.*, 1989, *3*, 299-309.
